# The role of far-field intravascular ultrasound in transcatheter aortic valve replacement

**DOI:** 10.1186/s43044-021-00227-9

**Published:** 2021-11-16

**Authors:** Ahmed Hassanin, Hasan Ahmad, Massoud Leesar, Diaa Hakim

**Affiliations:** 1 Department of Cardiology, Westchester Medical Center and New York Medical College, Valhalla, NY USA; 2 Department of Cardiology, University of Alabama at Birmingham, Birmingham, AL USA; 3 Intravascular and Cardiac Imaging Core Laboratory, Brigham and Women’s/Harvard School of Medicine, Boston, MA USA; 4 Department of Cardiology, Suez Canal University, Ismailia, Egypt

**Keywords:** Far-field intravascular ultrasound, Transcatheter valve replacement, TAVR, TAVI, Aortic stenosis, Aortic valve

## Abstract

Precise and accurate characterization of the aortic valve complex is a vital step in the procedure planning for transcatheter aortic valve replacement (TAVR). Far-field intravascular ultrasound (IVUS) is a novel technology that can be utilized to assess aortic valve annulus and predict paravalvular leak, with comparable results to multi-detector computed tomography—the current gold standard in the preprocedural planning in TAVR. Far-field IVUS carries the advantage of minimal contrast use and lower radiation exposure. In this commentary, we describe two cases of far-field IVUS use during TAVR procedures and review its role as a complementary tool to current the imaging modalities used in TAVR.

## Background

Transcatheter aortic valve replacement (TAVR) has revolutionized the treatment of patients with severe aortic stenosis. It has shown superior short and intermediate-term outcomes compared to the surgical aortic valve (SAVR) [1, 2]. Imaging modalities in TAVR are designed to accurately assess the aortic valve complex to select the appropriate size of the valve implant and create a vascular roadmap to identify potential obstacles to procedural success. Multi-detector computed tomography (MDCT) plays a central role in procedure planning by providing high spatial resolution of the valve morphology, dimensions, and pathological characteristics such as calcification, deployment angle, and anatomy of the peripheral arteries [3]. The role of transesophageal echocardiography (TEE) in TAVR has decreased over time. It is mainly limited to intraoperative imaging to assess valve positioning, expansion and detection of paravalvular aortic regurgitation (PVR) [4].

Intravascular ultrasound (IVUS) is a widely used imaging tool to guide coronary percutaneous interventions [5, 6]. IVUS imaging of complex coronary lesions and for stent optimization is recommended in chronic total occlusions and left main coronary artery interventions. Additionally, IVUS is utilized in peripheral interventions and is considered the gold standard imaging modality for measuring the luminal diameter of the aorta and selecting appropriate landing zones for endografts [7]. Recently published studies have reported the adaptation of a far field IVUS imaging technology to aortic valve disease. This includes sizing of the the aortic annuals, implant size selection and in predicting paravalvular regurgitation (PVR). This commentary report describes two cases of far-field IVUS use during TAVR procedures and reviews its role as a complementary imaging modality to MDCT and TEE.

## Cases

### Case 1

**Fig. 1:**
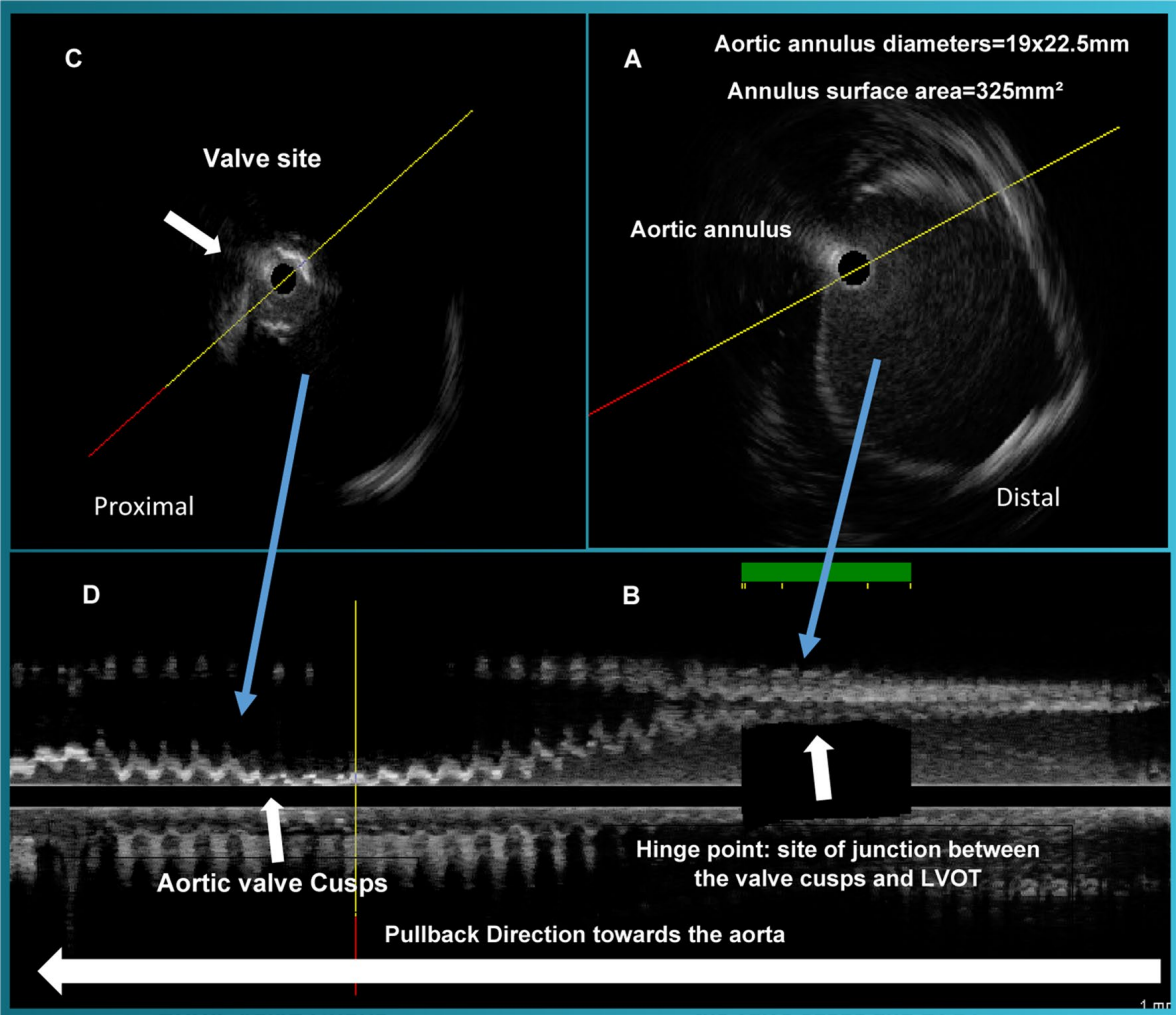
Intravascular ultrasound measurements of the aortic annulus diameters and surface area. The pullback is shown from right to left. **A** The IVUS image of the aortic annulus, the annulus area was 325 mm^2^; **B** corresponding longitudinal IVUS image at the site of junction between LVOT and the aortic cusps; **C** calcified aortic valve with signal drop-out; **D** corresponding longitudinal IVUS image of aortic valve cusps

An 89-year-old female with advanced emphysema and known severe calcific aortic stenosis presented for evaluation of chest pain and syncope. After excluding other etiologies for her presentation, aortic valve replacement was deemed appropriate. The patient’s society of thoracic surgeons (STS) risk score was 8.4%, making her a high-risk candidate for SAVR, so the patient was referred for TAVR evaluation. MDCT image quality was suboptimal due patient’s inability to hold her breath and movement artifact. Cardiac CT measurements revealed annular diameters of 19 × 24 mm and annulus surface area of 342 mm^2^ with a mean annular diameter of 21.5 mm. TEE was considered for further aortic annular assessment, but the patient’s advanced emphysema was a relative contraindication for sedation given the high risk for requiring endotracheal intubation. The decision was made to have the patient undergo intraprocedural aortic valve imaging by far-field IVUS using a 15 MH Atlantis catheter interfaced with i-Lab imaging systems (Boston Scientific, Natick, Massachusetts). After obtaining femoral arterial access, a pigtail catheter was advanced over the wire into the ascending aorta and placed at the non-coronary cusp soft tip. Subsequently, a straight wire was used to cross over the aortic valve to the left ventricle, and the catheter was then exchanged for an extra stiff wire. The IVUS catheter was advanced over the extra-stiff guidewire to the left ventricle. An automatic pullback IVUS run at a speed of 0.5 mm/s from the left ventricular outflow tract (LVOT) to the aortic root was undertaken. To enhance the visibility of the annulus and adventitial borders, the depth of IVUS penetration was increased to 50 mm and 2 mL agitated contrast media was injected through the lumen of the IVUS catheter. The cross-sectional area of the aortic valve annulus was obtained by planimetry of the circumference at the level of attachment of the aortic leaflet with the LVOT at its nadir point (most proximal to the LVOT). Figure 1 demonstrates reconstructed cross-sectional and longitudinal IVUS images obtained from the pullback of the IVUS catheter from the LVOT to the aortic root. This area is the hinge point. IVUS measurements for the minimum, maximum, average annular diameters and annular surface area were 19 mm, 22 mm, 20.5 mm, and 325 mm^2^, respectively, and correlated well with MDCT. A 23 mm Edward Sapien XT valve was successfully implanted with only trivial PVR. The post-procedure hospital course was uneventful, and the patient was discharged on post procedure day 3.

### Case 2

**Fig. 2:**
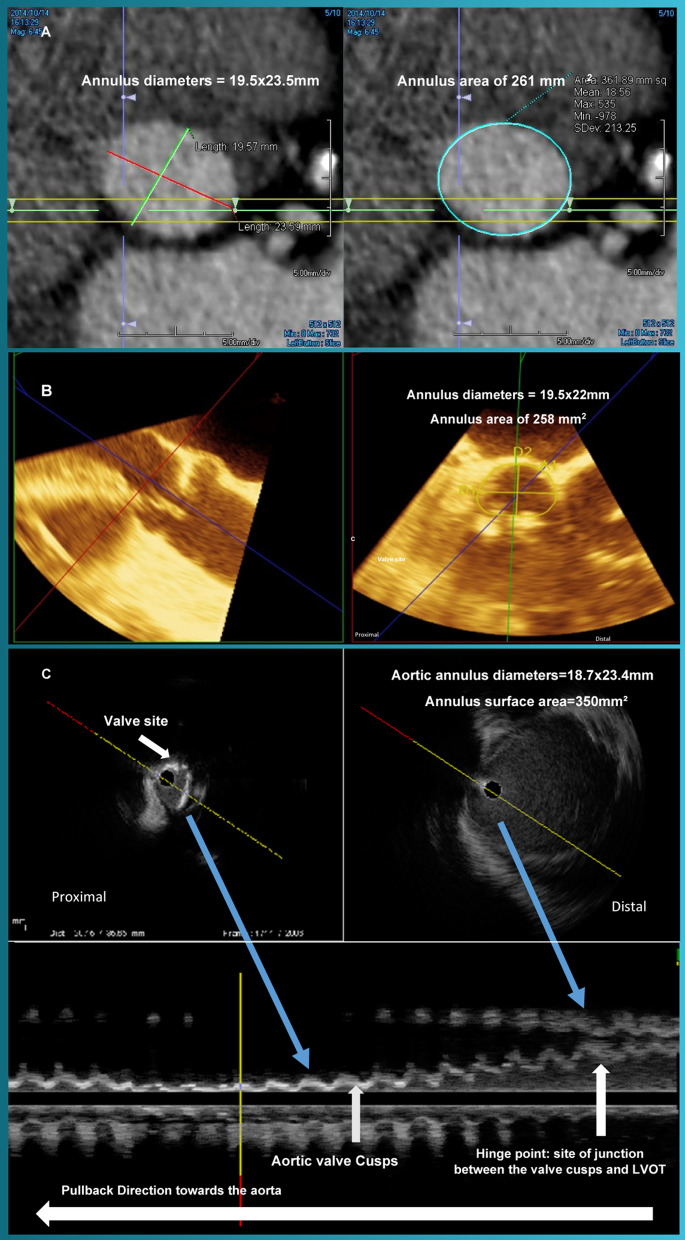
Comparative measurements of the aortic valve annulus obtained by MDCT, 3D TEE and far field IVUS. Panel **A** MDCT measurements of the aortic valve annulus; Panel **B** 2D TEE measurements of the aortic valve annulus; Panel **C** far field IVUS measurements of the aortic valve annulus

87-year-old female patient with hypertension and chronic kidney disease (CKD) stage IV presented with worsening New York Heart Association (NYHA) class III dyspnea symptoms. The patient was determined to have severe calcific aortic stenosis with an aortic valve area of 0.7 cm^2^, and a mean gradient of 42 mmHg on transthoracic echocardiogram. Her STS risk score was estimated to be 8.1%. After excluding other etiologies for the patient’s symptoms, TAVR was deemed appropriate. MDCT was done without contrast given the patient’s advanced CKD and the measurements revealed annular diameters of 19.5 × 23.5 mm, mean annular diameter of 22 mm, and annulus surface area of 361 mm^2^. During the patient’s pre TAVR left cardiac catheterization, angiography suggested moderate stenosis and calcification of both femoral arteries, so far-field IVUS was used to better assess the femoral arteries and demonstrated only mild stenosis. To further evaluate the aortic valve annulus, the IVUS catheter was then advanced over the wire to the aortic root. The IVUS measurements for the minimum, maximum, average annular diameters and annular surface area were 18.7 mm, 23.4 mm, 21.1 mm, and 346 mm^2^, respectively, similar to the measurement obtained on MDCT. TEE measurements obtained during the TAVR procedure showed an average annulus diameter of 19.5 and annulus surface area 358 mm^2^, which correlated well with measurements obtained by MDCT and IVUS. Figure 2 demonstrates comparative measurements of the aortic root obtained by the three imaging modalities. Given the anatomical evaluations provided by MDCT, TEE and IVUS, a 23 mm Edward Sapien XT was selected. The valve was successfully implanted with only trivial PVR. The patient’s hospital stay was without complications, and she was discharged on postoperative day 2.

## Discussion

MDCT is the gold stand for pre TAVR assessment of the aortic valve complex and assessing the iliofemoral vasculature for access consideration [3]. As compared to Two dimensional (2D) TEE, MDCT provides a more accurate assessment of the aortic annulus measurements and does not require sedation [4, 8]. The reliance on intraprocedural 2D TEE in TAVR has steadily declined over the past 5 years as conscious sedation became the standard approach. It has been associated with shorter hospital stay and a trend for lower mortality compared to general anesthesia [9]. Nevertheless, the quality of MDCT may occasionally be limited due to partial volume-averaging effects (blooming), heart/lung motion, arrhythmias, and patient motion [3], as was the case with the first patient presented. On the other hand, among patients with advanced CKD, the use of IV contrast for assessment of the iliofemoral arteries may be relatively contraindicated due to the increased risk of renal failure.

Far-field IVUS is a novel tool in the armamentarium of structural interventional cardiologists. Far-field IVUS has a maximum imaging diameter of 50 mm compared to a 20 mm diameter in traditional coronary IVUS catheters, which allows for adequate evaluation of the aortic valve complex. The use of IVUS in TAVR was first reported by Roy et al. in 2013 [10]. A case series in 2016 demonstrated an excellent correlation between aortic valve measurements obtained by MDCT and IVUS [11]. More recently, in 2017, Hakim et al. investigated the role of large-field IVUS vs. MDCT and 2D TEE for annular sizing and predicting PVR in 50 consecutive patients undergoing TAVR [12]. In their study, far-field IVUS demonstrated similar aortic annular diameters and area measurements compared to MDCT and performed as well in predicting PVR. There were strong correlations between IVUS and MDCT annular areas (*r* = 0.87, *P* < 0001) and mean diameters (*r* = 0.73, *P* < 0.0001). 2D TEE underestimated aortic annular diameter and did not correlate well with MDCT or IVUS. More recently, far-field IVUS use in TAVR has also been reported for the intraprocedural assessment of implanted valve frame geometry and leaflets mobility when under expansion is suspected [13].

Far-field IVUS has a strong correlation with MDCT and invasive angiography in the assessment of the iliofemoral arteries [14]. It carries the advantage of minimal contrast use and lower radiation exposure when performed at the- time of the routine pre-TAVR hemodynamic and coronary assessment as demonstrated in case number 2. Moreover, the data can be interpreted in real time, and are also a valuable adjunct to CTA in patients with borderline femoral access diameters or considerable CTA artifacts.

Far-field IVUS has a few limitations that should be acknowledged. If the IVUS catheter is not centralized, the images can be biased to one side, leading to oblique sections, and oversizing the aortic annulus. To avoid eccentric catheter position and motion artifacts, the IVUS catheter should be advanced over a stiff guidewire and measure the eccentricity index. If motion artifact is present the IVUS run can be repeated.

The IVUS catheter that was used during the cases was not primarily manufactured to visualize the aortic valve complex, so an IVUS catheter with better resolution can help in the assessment of the valve cusps, annulus, and PVR. The additional cost of utilizing the far-field IVUS catheter should also be considered.

## Conclusion

Far-field IVUS use in TAVR is a novel technology that could be used to assess aortic annulus and predict PVR. The technology can help assess leaflets' mobility when a valve under expansion is suspected. Far-field IVUS can also minimize contrast use in patients with advanced CKD who are undergoing a pre TAVR evaluation.

## Data Availability

Not applicable.

## Abbreviations

TAVR: Transcatheter aortic valve replacement
SAVR: Surgical aortic valve replacement
MDCT: Multi-detector computed tomography
TEE: Transesophageal echocardiography
PVR: Paravalvular aortic regurgitation
IVUS: Intravascular ultrasound
STS: Society of Thoracic Surgeons
LVOT: Left ventricular outflow tract
CKD: Chronic kidney disease
NYHA: New York Heart Association
2D: Two dimensional
